# Cultural adaptation and validation of the Survivor Unmet Needs Survey Short‐Form among cancer patients in China

**DOI:** 10.1002/nop2.720

**Published:** 2020-12-02

**Authors:** Tingting Yan, Wei Zheng, Dandan Wang, Wei Zhang

**Affiliations:** ^1^ The Nethersole School of Nursing The Chinese University of Hong Kong Hong Kong China; ^2^ The Second Affiliated Hospital of Zhengzhou University Zhengzhou China

**Keywords:** assessment, cancer, care needs, cross‐cultural, nurses. nursing, psychometrics, reliability, validity

## Abstract

**Aim:**

Cancer patients have long been found to have multiple types of unmet needs during their survivorship. Composite psychological instruments are essential for measuring the unmet needs of cancer patients. The objective of this study was to evaluate the psychometric properties of the Short‐Form Survivor Unmet Needs Survey (SF‐SUNS)‐Chinese version.

**Design:**

A cross‐sectional survey.

**Methods:**

The Chinese version was developed using the standard Functional Assessment of Chronic Illness Therapy (FACIT) translation methodology and 428 Chinese cancer patients participated in the survey between 2016‐2017. Inter‐rater reliability, exploratory factor analysis (EFA) and confirmatory factor analysis (CFA) were calculated.

**Results:**

Confirmatory factor analysis supported the four‐factor structure with good model fit. Cronbach's alpha of 0.894 for the overall scale and intra‐class correlation coefficients (0.869–0.884) indicated that reliability was satisfactory. The EFA extracted four factors with eigenvalues greater than 1 and these factors explained 50.68% of the total variance. The Chinese version of SF‐SUNS was confirmed to have the potential to become a useful and valid instrument. It could contribute to the assessment of unmet needs among Chinese cancer patients with accuracy and with respect to Chinese culture and context. This measurement of unmet needs may help promote cancer management and nursing quality. Clinical nurses and researchers could use the simple assessment tool to target the individual needs of Chinese cancer patients and then provide more personalized care efficiently.

## 1 INTRODUCTION

Cancer treatment is progressing relatively rapidly due to improvements in treatment protocols and preventive health care. The 5‐year relative survival rate for all cancers has increased 20–23 percentage points over the past three decades, and the decline in cancer death rates is dramatically (Miller et al., 2016). Meanwhile, the increasing incidence of cancer had become a major public health problem, which had made many cancer patients becoming an increasingly heavy burden in many countries. The incidence of cancer in China is steadily increasing, from 220 million in 2006 to a projected 550 million in 2020 (Bray et al., 2018). It is predicted that 36.9% of Chinese cancer patients will survive at least 5 years after diagnosis around 2015; cancer is a significant public health problem in China (Chen et al., 2016). Due to the deterioration of health status, the treatment burden and various cancer symptoms, cancer patients have multiple kinds of needs that are not being met to their satisfaction during survivorship. Although the average survival period has been extended, long‐term cancer experience affected diverse aspects of patients that may differ from patient to patient, necessitating individualized interventions based on each person's current unsatisfied needs (Houts et al., 1986). The unmet need of cancer patients had received widespread attention. Unmet needs were generally defined as “the needs that patients have not been satisfied” or “the discrepancy of needs and service received”. It can be confirmed that the “needs” are what do cancer patients want, but the “unmet needs” are that what do cancer patients want help with additional attention and support after getting treatment or care. Unlike various needs measurement and other clinical outcomes, unmet needs provide an opportunity to investigate the disparity between patients' concerns and the level of assistance they require (Campbell et al., 2009; Sanson‐Fisher et al., 2000), which contribute significantly to patient‐centred care (Tzelepis et al., 2015).

Reducing the negative impact of cancer experiences requires better insights about cancer patients' needs and optimization of service provision against needs (Hall et al., 2015). To avoid treatment and nursing inefficiencies, it is important to have early and accurate detection of patients’ own concerns, that is, their unmet needs (Boyes et al., 2009; Mühlan et al., 2008). In a multicenter, large sample study, unmet information needs of 4,020 cancer patients were prevalent in 36%–48%, patients who had more unmet needs reported more anxiety, depression and lower quality of life (Faller et al., 2016). 10%–24% of patients had at least one psychosocial unmet need without considering the distress level (Van Scheppingen et al., 2011). Moreover, it had been found that cancer treatment and symptom burden affected patients' quality of life through unmet needs in their survival time; the unmet needs mediate the relationship between the above (Cheng et al., 2016). Unmet needs of cancer patients acted as a moderator when it decreased patient perception of physician empathy (Lelorain et al., 2015). According to previous studies, although cancer patients' unmet needs are diverse and heterogeneous, it can be categorized into several domains (Effendy et al., 2015; Rowlands et al., 2015; Willems et al., 2016). Moreover, unmet needs of cancer patients might significantly differ between countries due to the difference of culture, personal values and economics. Thus, developing an instrument or evaluate a Chinese version with strict structure to measure unmet needs is of very importance for both academic study and practical application in China.

## 2 BACKGROUND

Quality of life (Cossich et al., 2004) and patient satisfaction (Almeida et al., 2015) measures are widespread in cancer care, while they fail to indicate whether these decrements are problematic to patients, or require service input. Identifying unmet needs, which may contribute to improvement of quality of life, enables more individualized and potentially cost‐effective targeted care. Therefore, a standardized measurement that is specific to assessing the unsatisfactory needs of cancer patients would be considered important and meaningful. The clinical application of a standardized measurement could provide a way for understanding patterns of problems and individual unmet needs across patients, which is beneficial for improving cancer patients' treatment compliance and quality of life.

Several valid and reliable questionnaires assess the unmet needs of cancer patients. However, few of them have widely validated Chinese versions that have sound psychometric properties (Moghaddam et al., 2016). Commonly used scales for evaluating unmet needs include the Survivor Unmet Needs Survey (SUNS), the Supportive Care Needs Survey (SCNS) and the Cancer Survivors Supportive Unmet Needs (CaSUN) measure.

The Survivor Unmet Needs Survey (SUNS) is a standardized measurement for cancer patients. It showed good psychometric properties with acceptable test–retest reliability and internal consistency (all Cronbach's alphas above 0.90) (Campbell et al., 2011). What makes it special from other cancer‐specific unmet needs measuring tools (Boyes et al., 2009; Hodgkinson et al., 2007) is that the SUNS was developed and evaluated in a population‐based sample, which proved that the contents and entries of the scale are sufficient. It had been used in several cross‐cultural and trans‐regional studies to assess the current unmet concerns in cancer patients (Hall et al., 2013, 2015). The Short‐Form Survivor Unmet Needs Survey (SF‐SUNS) was developed from the SUNS using a heterogeneous sample of 1,589 cancer patients. The applicability of the scale for using in clinical practice had been strengthened (Campbell et al., 2014; Taylor et al., 2016). The 60‐item Supportive Care Needs Survey (SCNS) has five subscales (psychologic needs, health system and information, physical and daily living, patient care and support and sexuality needs); Cronbach's alphas 0.87–0.97 (Bonevski et al., 2000). However, SCNS focuses on short‐term cancer survivors, where even the SCNS module was developed specifically for breast cancer survivors 6–24 months post‐treatment (Thewes et al., 2004). The 34‐item short‐form SCNS‐SF34, translated and validated for the Chinese cancer population (Au et al., 2011), is reliable but not a cancer survivor‐specific questionnaire (Han et al., 2017) and therefore unsuitable for extensive applications in the Chinese studies. The CaSUN (Keeman et al., 2018) is survivor‐specific questionnaire. While the CaSUN has a relatively large quantity of items on quality of life, it lacks items on financial issues. In mainland China, there is a lack of appropriate and population‐based scales to evaluate the multidimensional unmet needs in cancer survivorship among Chinese cancer patients.

Considering that there is no particular population‐based scale in China to assess unmet needs in cancer survivorship, the purpose of this study was to cultural adapt and validate the Chinese version of SUNS‐SF (SUNS‐SFC) for Chinese cancer patients about unmet needs through a cross‐sectional survey. Culturally sensitive instruments measuring unmet needs are essential to enable researchers and nurses to understand gaps between clinical cancer care and patient needs.

## 3 METHODS

### 3.1 Study design and sample

This was a cross‐sectional study using a panel of clinical and psychological experts and the cancer patients’ recruitment was conducted in a provincial hospital in China. The study included two phases. First, the SUNS‐SF—developed for the Australian cancer patient population—was independently translated from English into simplified Chinese following standard translation methodology. Second, main crucial psychometric properties of Chinese version of SUNS‐SF (SUNS‐SFC) were assessed. The sample size is calculated based on the desired size to meet the recommendations of a ratio of 10 respondents per item for the psychometric assessment (MacCallum et al., 1996). This study recruited 428 cancer patients during the investigating period between 2016–2017. Eligible participants meet the following requirements: (a) had a histologically confirmed diagnosis and were known to have suffered cancer at least 12 months prior to data collection; (b) were aged above 18 years old, (c) were native Chinese and proficient in Mandarin communication; (d) agreed to participate in the research and offered a written consent about their voluntary and (e) were able to comprehend and complete the questionnaires. Cancer patients who had previously expressed reluctance to participate in this research were excluded from the survey. Cancer patients who were suffering from other or multiple life‐threatening diseases were excluded from the survey.

### 3.2 Measurements

#### 3.2.1 Short‐Form Survivor Unmet Needs Survey (SF‐SUNS)

The 30‐item SF‐SUNS is composed of four dimensions to evaluate unmet needs in the last month that relate to finding information, medical care and psychological health (Campbell et al., 2014). Each item used a 5‐point Likert scale system: “no unmet need” to “very high unmet need,” with a score from 0–4. The value range of the overall score is between 0–120, which is the sum of all the 30 items' scores constitutes. Higher scores represent more unmet needs and worse unsatisfactory on the relevant survivor concerns (Campbell et al., 2014). The SUNS‐SFC demanded about 10 min to accomplish.

#### 3.2.2 Functional Assessment of Cancer Therapy‐General scale (FACT‐G, version 4)

The FACT‐G (version 4) is a cancer‐specific core measurement for addressing the level of quality of life. Its Chinese version has been applied in China for many years. It consists of 27 items and all entries formed four domains of the scale related to cancer patients' physical, emotional, social, family and functional aspects, scoring from 0–4. Compared with other standardized quality of life survey tools developed for cancer patients, FACT‐G has greater discrimination and requires smaller sample size, with less response burden (Cheung et al., 2005).

#### 3.2.3 Patient Health Questionnaire 9

The Patient Health Questionnaire 9 (PHQ‐9) is used to assess the symptoms of depression through requesting participants to define the frequency and the degree of their emotional distress in the past 2 weeks (Chen et al., 2010). This 9‐item 4‐point Likert scale ranged from 0 (not at all)–3 (nearly every day). The PHQ‐9 has been found to be a highly acceptable and valid depression diagnostic and severity instrument (Feng et al., 2016).

### 3.3 Procedure

After the research team discussed a variety of widely used classical translation methods, we finally decided to adopt the standard Functional Assessment of Chronic Illness Therapy (FACIT) translation methodology (Eremenco et al., 2005), a method for successfully producing universal translations of self‐reported instrument used in multiple countries worldwide, to accomplish the Chinese translation work of the SF‐SUN. It ensured that the translation procedures are exhaustive and rigorous and less bias (Debb et al., 2011; Qiao et al., 2017).

The process included forward translation, reconciliation, back‐translation, quality control, independent reviews, finalization process and pre‐testing. First, two native Chinese translators (one was translation expert and the other was epidemiologist) translated the original version into the simplified Chinese version. Second, a third independent Chinese oncologist who had not browsed the scale before nor attended in the forward translation to choose a better one from the two versions and compare, check, resolve discrepancies between them to modify it into a coordinated version. Third, one English translation expert who was proficient in Chinese and was not involved in the previous steps translated the reconciled Chinese version into English. Fourth, the second back‐translator who was a native English speaker provided a translation with capturing the literal meaning of each item through simple language. Both were unaware of the original English source items. Fifth, the second author of our study assessed the equivalence of the original English version and the two back‐translations, adjusted and harmonized the items in question. The further refinement was based on the independent interview of three diglossia specialist (one psychological expert and two cancer rehabilitation professionals), including analysing all versions and our discussion comments. After that, a coordinator completed the final version and sent it to two independent proofreaders for final grammatical formatting.

Finally, 60 Chinese cancer patients who met the inclusion criteria participated in the pre‐test of the integrated version. They were interviewed about the items that they feel confusing or uncomfortable. Then, they were asked to reframe the items if necessary. Hence, after considering the experiences and opinions of the patients who participated in the survey, we determined our final SUNS‐SFC.

### 3.4 Data collection

Our unified‐trained interviewers (three nursing postgraduate students) have ensured that all participators completed our research survey. They helped collect demographic information and clinical characteristics of the participants from the electronic medical records and responsible doctors. Eligible participants offered the informed consent. After that, an interview that focused on filling confusion and checking whether there are missing contents was conducted.

### 3.5 Ethical considerations

The Ethical Committee of the affiliated hospital of University approved the protocol of this study (institutional review board no. 2016N011). The study researchers got permission for access to the medical records of the patients. Eligible patients were approached at their regular medical consultations. Before the study, participants signed informed consent about their voluntary. The consent form was obtained, and all data were kept confidential and anonymous. Eligible patients were fully informed about the study purpose and data confidentiality. They were also informed the rights to refusal and uncontested withdrawal.

### 3.6 Data analysis

To analyse and summarize the demographic and clinical characteristics, we used descriptive statistics. In this study, we estimated internal consistency reliability by Cronbach's alpha (Bland & Altman, 1997). The retest was conducted by asking 60 participants to complete the survey for the second time after 3 weeks of the first‐time response. The intra‐class correlation coefficients (ICCs) were adopted to calculate test–retest reliability (Landis & Koch, 1977). As for content validity, we invite a panel of experts to evaluate the translation equivalence and cultural compatibility of each item from SUNS‐SFC, a value of 0.8 or above is considered as the satisfactory level of agreement. Regarding convergent validity, we investigated the correlations between the SUNS‐SFC and other validated psychosocial measures including FACT‐G and PHQ‐9 with Pearson correlation coefficient.

Exploratory factor analysis (EFA) provides a relatively rigorous replication test because it does not specify a predetermined factor solution and the data‐driven approach of EFA seems advisable. Therefore, the construct validity of SUNS‐SFC was evaluated using exploratory factor analysis (EFA) with maximum‐likelihood extraction and varimax rotation. Confirmatory factor analysis (CFA) was performed to validate the factorial validity of the SUNS‐SFC to verify whether the prior structure of the original survey applies equally to our study. To estimate the degree of model‐data fit, we used fit indices including degrees of freedom (*χ* ^2^/*df*), comparative fit index (CFI), Tucker–Lewis index (TLI), root‐mean‐square residual (RMR), root‐mean‐square error of approximation (RMSEA) and other fitting indicators. The indices outcome with *χ* ^2^/*df* between 1.0–3.0, CFI, TLI greater than 0.90, RMSEA less than 0.08, indicated that the model fit is apposite; and with 1 < *χ* ^2^/*df* < 2.0, CFI, TLI > 0.95, RMSEA < 0.05, suggested that the model meets the more stringent fitness criteria.

The above statistical analyses were conducted using IBM SPSS 20.0 and MPLUS version 7.31 (Muthén & Muthén, 2012). The statistical significance of two‐sided was set at the level of *p* < .05 for all statistical tests.

## 4 RESULTS

### 4.1 Sample characteristics

We included a total of 450 eligible cancer patients and 428 (95.1%) finally gave us completed questionnaires with attached informed consent. More than half of our study were female (60.3%). The main types of cancer in our study were gynaecological cancer, breast cancer, digestive system cancer (stomach cancer, oesophageal cancer) and lung cancer, of them 268 (62.6%) staged I or II in the TNM system. Concrete characteristics data and clinical information of participants are shown in Table 1.

**TABLE 1:** Demographic Information and Clinical Data (*N* = 428)

Characteristics	Number (%)
Age (years)	
Mean ± *SD*	55.25 ± 13.4
Range	23–87
Age < 60	283 (66.1)
Age > 60	145 (33.9)
Gender	
Male	170 (39.7)
Female	258 (60.3)
Ethnicity	
Han	407 (95.1)
Others	21 (4.9)
Marital status	
Married	394 (92.1)
Single	6 (1.4)
Divorced or widowed	28 (6.5)
Occupation	
Employed	195 (45.6)
Unemployed	233 (54.4)
Education states	
Primary school and below	73 (17.1)
Junior school	162 (37.9)
Senior high school	111 (25.9)
Bachelor and above	82 (19.2)
Perceived Income	
Low	36 (8.4)
Middle	204 (47.7)
Good	120 (28.0)
High	68 (15.9)
Type of cancer	
Cervical cancer	118 (27.6)
Lung cancer	73 (17.1)
Breast cancer	57 (13.3)
Stomach cancer	79 (18.5)
Colorectal cancers	39 (9.1)
Oesophageal cancer	28 (6.5)
Thyroid cancer	18 (4.2)
Prostatic cancer	16 (3.7)
Stage of disease	
I/II	268 (62.6)
III/IV	160 (37.4)
Time since diagnosis (years)	
Mean ± *SD*	1.7 ± 1.2
Adjuvant therapy	
Only had chemotherapy	337 (78.7)
Had chemotherapy and radiation therapy	91 (21.3)
Family history of cancer	
Yes	7 (1.6)
No	417 (98.3)

### 4.2 Reliability

The Cronbach's *α* coefficient for the overall scale was 0.894, indicating that it has great internal consistency. Simultaneously, Cronbach's *α* coefficients of four subscales were in the optimum range and the “Unmet coping, sharing and emotional Needs” dimension demonstrated good internal consistency (Cronbach's *α* = 0.812), the “Unmet work and financial needs” dimension showed slightly worse, but still acceptable internal consistency (Cronbach's *α* = 0.702). In our results, the test–retest reliability correlation coefficients of four domains were all greater than 0.8, which ranged from 0.869–0.884. As for the intra‐class correlation coefficient (ICC), it was 0.916 for the total scale. The results are given in Table 2.

**TABLE 2:** Internal consistency and test–retest reliability of the SUNS‐SFC

Domains (Items number)	Cronbach's Alpha coefficients (*N* = 428)	Intra‐class correlation Coefficient (*N* = 60)
Unmet information needs (3)	0.713	0.872
Unmet work and financial needs (8)	0.702	0.879
Unmet needs for access and continuity of care (6)	0.703	0.869
Unmet coping, sharing and emotional needs (13)	0.812	0.884
Overall scale^a^ (30)	0.894	0.916

Abbreviation: SUNS‐SFC, Chinese version of the Short‐Form Survivor Unmet Needs Survey.

^a^ Based on 30 items.

### 4.3 Content validity

All the nine experts gave the SUNS‐SFC their objective appraisal and most of them had more than 6 years of clinical and research experiences in cancer care or treatment. The experts agreed that the SF‐SUNS showed the utmost solicitude for the patients' concerns, which is particularly designed to measure unmet needs. Moreover, they hold that SUNS‐SFC is relatively succinct to be completed when compared with a variety of complex and lengthy needs questionnaire. The item‐level CVI ranged from 0.89–1.0 and the scale‐level CVI reached 0.91, which indicated the scale has excellent content validity.

### 4.4 Construct validity

#### 4.4.1 Exploratory factor analysis

Construct validity was evaluated by exploratory factor analysis (EFA). The assumptions about the applicability for factor analysis were examined. The Kaiser–Mayer–Olkin (KMO) measure was equal to 0.931 (*p* < .001); Bartlett's test of sphericity with *χ* ^2^ = 3,612.81 was significant (*p* < .001), which showed that the data were suitable for factor analysis. In terms of factor selection, the criteria include the eigenvalue >1 and the variance percentage explained by the factor is higher than 5%. Using maximum‐likelihood extraction and varimax rotation, we extracted four components that explained a total variance of 50.68%. The four‐factor structure of the model was the same as the original scale (Campbell et al., 2014), which was consistent with the original four dimensions. The factor loadings for the four factors ranged from 0.468–0.748, where almost all were higher than 0.5. All the results are shown in Table 3.

**TABLE 3:** Factor loadings of Exploratory Factor Analysis with 30 unmet needs items of the SUNS‐SFC

Items on factor	Unmet information needs (factor 1)	Unmet work and financial needs (factor 2)	Unmet needs for access and continuity of care (factor 3)	Unmet coping, sharing and emotional needs (factor 4)
Finding information about complementary or alternative therapies	0.555	0.269	0.178	0.278
Dealing with fears about cancer spreading	0.748	0.229	0.103	0.146
Dealing with worry about whether the treatment has worked	0.728	0.229	0.213	0.231
Worry about earning money	0.310	0.585	0.176	0.139
Having to take a pension or disability allowance	0.385	0.517	0.189	0.076
Paying household bills or other payments	0.312	0.536	0.236	0.176
Finding what type of financial assistance is available and how to obtain it	0.028	0.587	0.197	0.095
Finding car parking that I can afford at the hospital or clinic	−0.003	0.690	0.163	0.107
Understanding what is covered by my medical insurance or benefits	0.168	0.692	0.039	0.195
Knowing how much time I would need away from work	0.277	0.531	0.042	0.222
Doing work around the house (cooking, cleaning, home repairs, etc.)	0.145	0.529	0.222	0.223
Having access to cancer services close to my home	0.130	−0.038	0.706	0.113
Getting appointments with specialists quickly enough (oncologist, surgeon, etc.)	0.331	0.277	0.516	0.265
Getting test results quickly enough	0.243	0.307	0.624	0.151
Having access to care from other health specialists (dieticians, physiotherapists, occupational therapists)	0.141	0.333	0.597	0.295
Making sure I had enough time to ask my doctor or nurse questions	0.280	0.195	0.520	0.369
Getting the healthcare team to attend promptly to my physical needs	−0.018	0.240	0.673	0.235
Telling others how I was feeling emotionally	−0.123	0.278	0.132	0.591
Finding someone to talk to who understands and has been through a similar experience	0.310	−0.014	0.137	0.597
Dealing with people who expect me to be “back to normal”	0.153	0.262	0.150	0.531
Dealing with people accepting that having cancer has changed me as a person	0.040	0.076	0.028	0.589
Dealing with reduced support from others when treatment has ended	0.239	0.151	0.314	0.548
Dealing with feeling depressed	0.285	0.093	0.180	0.530
Dealing with feeling tired	0.184	0.246	0.163	0.468
Dealing with feeling stressed	0.053	0.064	0.184	0.572
Dealing with feeling lonely	0.120	0.114	0.199	0.512
Dealing with not being able to feel “normal”	0.314	0.165	0.233	0.570
Trying to stay positive	0.350	0.074	0.211	0.491
Coping with having a bad memory or lack of focus	−0.067	0.166	0.251	0.575
Dealing with changes in how my body appears	0.193	0.169	−0.055	0.585
Eigenvalues (after rotation)	2.731	3.664	3.018	5.791
% of variance explained (total = 50.681%) (after rotation)	9.103	12.214	10.062	19.302

#### 4.4.2 Confirmatory factor analysis

The confirmatory factor analysis (CFA) was performed on the sample of 428 participants to test the four‐factor model fit of SF‐SUNS, which had been verified by previous studies.

Results of the CFA: (1) *χ* ^2^ = 530.006, GFI = 0.926, AGFI = 0.914, RMR = 0.027, Standardized RMR = 0.041, RMSEA = 0.028; (2) IFI = 0.954, TLI = 0.949, CFI = 0.953; (3) PGFI = 0.795, PNFI = 0.768, *χ* ^2^/*df* = 1.328, HOELTER (CN) = 360 > 200.

**FIGURE 1:**
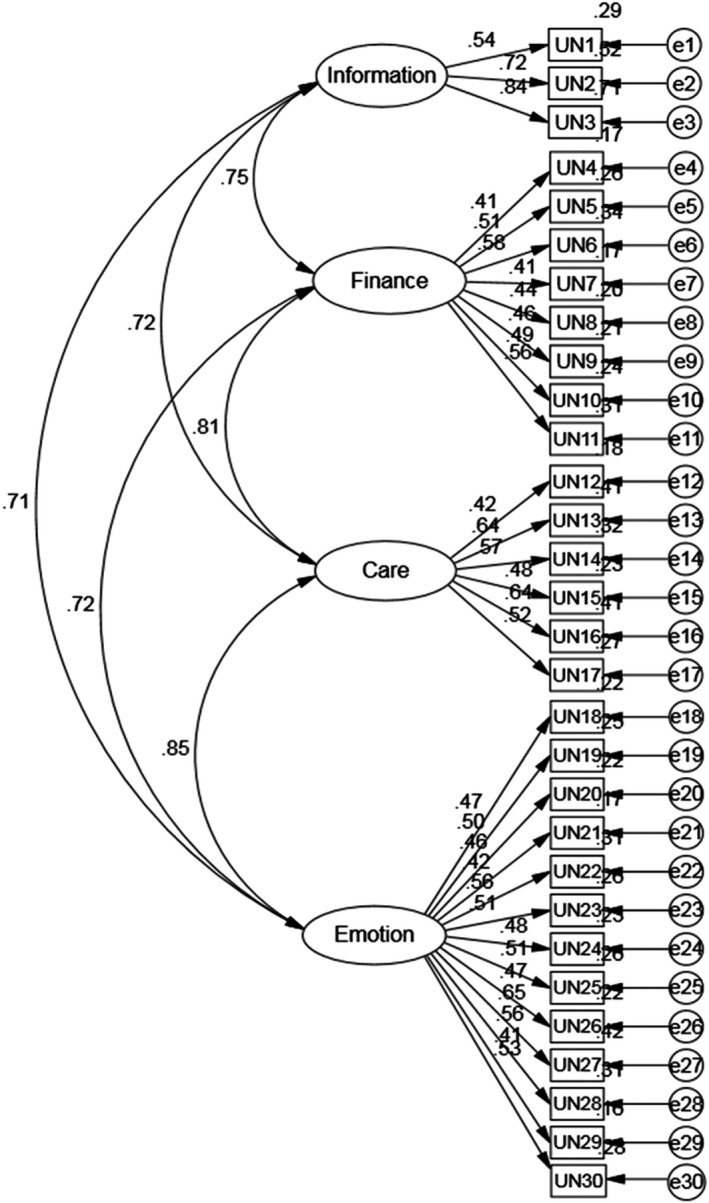
Path diagram of a confirmatory factor analysis of SUNS‐SFC. The values correspond of the standardized estimates

The above data results indicated that there were goodness‐of‐fit indices of the model and standardized estimates of the model are shown in Figure 1. Thus, the thirty‐item four‐factor correlated model is acceptable. This can be used as the basis for the following subsequent reliability and validity assessment in our study.

### 4.5 Convergent validity

In addition, for the testing of convergent validity, the findings revealed that the SUNS‐SFC had significant positive relationships with the self‐rated PHQ‐9 (*r* = 0.692, *p* < .01) and had negative correlation with FACT‐G (*r* = −0.642, *p* < .01, correlation coefficient (*r*) of each dimension with the scale ranged from −0.570––0.623). The results demonstrated acceptable convergent validity of the measurement.

## 5 DISCUSSION

In a sample of Chinese cancer patients, our study described culture debugging process and rigorous psychometric testing of the SUNS‐SFC questionnaire. Although differing from the English‐speaking patients in cultural backgrounds and lifestyle, the concerns of unmet needs for cancer satisfaction still concentrated on similar areas.

In our study, the SUNS‐SFC had been tested the reliability, factorial validity, content validity, convergent validity. And most results of the psychometric indicators were great. About the reliability analysis, we found that the Cronbach's *α* value of the overall scale was 0.894, with the Cronbach's α coefficient of each dimension ranged from 0.702–0.812, which is in parallel to the results of previous studies (Campbell et al., 2014; Hall et al., 2013). Satisfactory reproducibility had been proved by test–retest reliability analysis with the result that each intra‐class correlation coefficient of four dimensions was higher than 0.8. Notably, compared with other domains, the intra‐class correlation coefficient (ICC) value for “Third dimension: Unmet needs for access and continuity of care” were relatively low. This might be attributable to the situation that cancer patients in China were less sensitive to the unmet care needs of continuity of care in the future.

In the EFA, we found that the original four‐factor structure and measurement model was supported by our study. The EFA results confirmed the adequate fit of the SUNS‐SFC to Chinese patients with cancer. Moreover, the results of EFA indicated the survey have the four dimensions, which is consistent with the original scale. It was shown that four components explained a total of 50.68% of the variance and the factor loadings were satisfactory. The above data supported our conclusion after compared with other previous unmet needs survey validating studies (Boyes et al., 2009; Campbell et al., 2011).

In the CFA of testing construct validity of the SUNS‐SFC, the original four‐factor structure and measurement model was supported by results. The root‐mean‐square error of approximation (RMSEA) was <0.05, better than the standard requirement of <0.08. Furthermore, with the evidence that both root‐mean‐square residual (RMR = 0.027) and standardized RMR (0.041) were <0.05, we could draw the model to a “reasonable fit” conclusion. Moreover, GFI, AGFI and TLI values > 0.90 and remarkably, IFI, CFI values > 0.95, the above fitting data support our conclusion.

Since validity is the psychometric property with a relatively greater reference value, which shows how accurately the scales measure the quality of its desired estimates, we conducted content validity and convergent validity. The item‐level CVI ranged from 0.89–1.0 and the scale‐level CVI reached 0.91. These illustrated that the experts reached the broad consensus on their opinions about the good content of the SUNS‐SFC. Regarding convergent validity, significant positive relationships with the self‐rated PHQ‐9 (*r* = 0.692, *p* < .01) and negative correlation with FACT‐G demonstrated the acceptable convergent validity of the SF‐SUNS.

In general, these findings should enable researchers to refine their care strategies and target different groups to make more efficient use of healthcare resources and develop interventions that are sustained across time due to the inclusion of tailored characteristics. Furthermore, a possible intervening measure that can be used now to decrease unmet needs in cancer patients is the procedural use of SUNS‐SFC survey before and/or during the clinical encounter. In our clinical investigation, this survey has shown to enhance patient participation during initial oncology nursing. Moreover, the clinical application of SUNS‐SFC could help medical staffs take timely and effective care and may hold promise of improving the quality of life of cancer patients.

### 5.1 Limitations

There were still some limitations to our current study. First, although our participants were from cities and villages in central China, the current sample may not be enough to represent the entire country. Second, response to unmet needs was based on cut points from our cross‐sectional survey, a longitudinal research would be needed. Finally, we did not assess the discriminate validity. We suggest that future studies measure it and reconfirm the factorial validity of the SUNS‐SFC. In addition, we suggest that more research may continue before the scale is widely used in clinical nursing practice. Such needs assessment could be a useful way to effectively reallocate care resources and improve the quality of cancer care.

## 6 CONCLUSIONS

A unique contribution of our study is that we found the SUNS‐SFC was appropriate and valid in measuring unmet needs after translation and intercultural adaptation. The scale could help improve the oncology rehabilitation efficacy of Chinese patients. This study will add to growing evidence about the appropriateness and the applicability of the SUNS‐SFC in a variety of cultural environments. The unmet needs of cancer patients have great reference value for upgrading management of medical needs and for making individualized targets to improve their physical and mental status. This measurement of unmet needs may help improve cancer care and quality of nursing in China. Future longitudinal and multicenter study is recommended to examine unmet needs over different time spans and different cancer populations.

## CONFLICT OF INTEREST

The authors declare no conflict of interest.

## AUTHOR CONTRIBUTIONS

W. Zheng and T.T.Y. made concept and design; D.D.W, T.T.Y. and W. Zhang collected the data; T.T.Y. and W. Zheng analysed the data; D.D.W, T.T.Y. and W. Zheng prepared the manuscript; and T.T.Y. and W. Zheng reviewed the manuscript All authors substantially contributed to the revising and final approval of the article.

## ETHICAL APPROVAL

The Ethical Committee of the Second Affiliated Hospital of Zhengzhou University approved the protocol of this study. Eligible patients were approached at their regular medical consultations. The consent form was obtained, and all data were kept confidential and anonymous.

## Data Availability

The data sets used and/or analysed during the current study are available from the corresponding author upon reasonable request.
